# Grating Couplers on Silicon Photonics: Design Principles, Emerging Trends and Practical Issues

**DOI:** 10.3390/mi11070666

**Published:** 2020-07-08

**Authors:** Lirong Cheng, Simei Mao, Zhi Li, Yaqi Han, H. Y. Fu

**Affiliations:** Tsinghua-Berkeley Shenzhen Institute (TBSI), Tsinghua University, Shenzhen 518000, China; clr18@mails.tsinghua.edu.cn (L.C.); maosm19@mails.tsinghua.edu.cn (S.M.); li-z19@mails.tsinghua.edu.cn (Z.L.); Sherry_FTO@outlook.com (Y.H.)

**Keywords:** silicon photonics, grating couplers, integrated waveguide devices

## Abstract

Silicon photonics is an enabling technology that provides integrated photonic devices and systems with low-cost mass manufacturing capability. It has attracted increasing attention in both academia and industry in recent years, not only for its applications in communications, but also in sensing. One important issue of silicon photonics that comes with its high integration density is an interface between its high-performance integrated waveguide devices and optical fibers or free-space optics. Surface grating coupler is a preferred candidate that provides flexibility for circuit design and reduces effort for both fabrication and alignment. In the past decades, considerable research efforts have been made on in-plane grating couplers to address their insufficiency in coupling efficiency, wavelength sensitivity and polarization sensitivity compared with out-of-plane edge-coupling. Apart from improved performances, new functionalities are also on the horizon for grating couplers. In this paper, we review the current research progresses made on grating couplers, starting from their fundamental theories and concepts. Then, we conclude various methods to improve their performance, including coupling efficiency, polarization and wavelength sensitivity. Finally, we discuss some emerging research topics on grating couplers, as well as practical issues such as testing, packaging and promising applications.

## 1. Introduction

Integrated photonics is a promising solution to provide low-cost and high-performance photonic devices and systems, while complementary metal oxide semiconductor (CMOS) compatible silicon photonics such as silicon-on-insulator (SOI) platform has emerged as one of the most important technology for next generation on-chip optical interconnects [1,2,3,4]. Various optical components are already implemented on silicon photonics, including on-chip light source [5], high speed modulators [6], wavelength division multiplexers and optical switches [7]. Apart from datacom [8], silicon photonics has also found its applications in biosensing [9] and light detection and ranging (LiDAR) [10]. Although there are many high-performance optical components already available on SOI, one key challenge encountered by silicon photonic chip is to couple light to and from optical fibers efficiently. The standard fiber for datacom is single-mode fiber (SMF), which has a mode field diameter (MFD) of near 10 μm at 1550 nm. Efficient coupling from SMF to waveguide with size of hundreds of nanometers is a challenge due to the large modal size mismatch. This problem is usually addressed using two solutions, in-plane (butt) edge coupling and off-plane (vertical) grating coupling [11,12,13].

For edge coupling, fiber is placed at the chip facet and aligned with on-chip waveguide horizontally. Edge couplers usually consist of an inverse tapered waveguide, which means the width of waveguide is gradually decreasing along the propagation of light to the edge [14]. As it gradually decreases, light confinement is reduced and the mode size becomes larger to be comparable with fiber mode. It is reversed for fiber-to-chip propagation case, where Gaussian fiber mode distribution will be confined in the tapered waveguide as its size gradually increase. Edge couplers can achieve high coupling efficiency (CE), large bandwidth (BW) and low polarization dependent loss (PDL) [11]. However, a properly cleaved and polished facet with strict smoothness requirement is needed to reduce the loss, which adds extra fabrication cost considering mass manufacturing. The high performance also comes with reduced flexibility, since they have relatively large footprint and must be placed at the edge of chip. In addition, edge coupling solutions have low fiber-chip alignment tolerance and prohibit wafer-level testing, which will further increase the cost per chip and require higher accuracy for testing and packaging.

On the contrary, vertical grating couplers (GCs) are much more flexible in terms of arbitrary coupling position on chip, compact size, easy fabrication and wafer-level testing capability. For grating coupling, fiber is placed above diffractive grating structures on the top of chip. A vertical GC changes the off-plane wave-vector direction of light to the in-plane waveguide direction, and then couples the light into waveguide using a spot-size converter. Although GCs have the above-mentioned advantages, they also have some drawbacks coming with their operation principles. First, they usually have lower CE compared with edge couplers, while numerous research works have focused on improving CE of GC. Secondly, gratings are intrinsically sensitive to both wavelength and polarization. We first introduce basic working principles and theories for GCs, and then we review specific design principles that are widely adopted to overcome these limitations. In addition, we also discuss some emerging research trends in silicon PIC that also apply to GC design, including new functionalities, new design methodology and new material platforms in recent years. Finally, practical issues concerning testing, packaging and applications of GC are also provided.

## 2. Fundamentals of Grating Coupler

**Figure 1:**
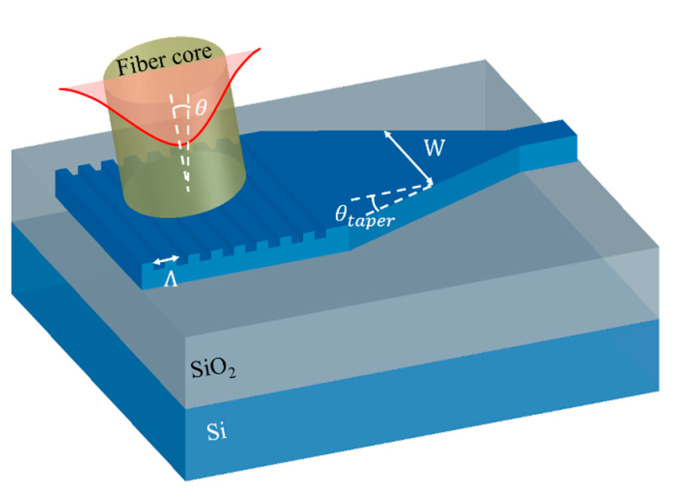
Schematic structure for a 1D grating coupler (GC) with linear waveguide taper and key parameters: period Λ , fiber tilt angle *θ*, varying taper width W and taper angle $\theta_{taper}$ .

Grating is basically a varying arrangement of different materials or structures on certain surface, usually periodic. On SOI-based photonic chips, it is realized through etching on SOI or selectively depositing amorphous silicon on SOI. Either etching or deposition creates refractive index variation. If the index variation has a period larger than the wavelength of light inside the grating material, diffraction effect prevails. Otherwise, the light propagation in grating will exhibit similar feature as uniform medium, which becomes more significant as its period decreases [15,16,17]. GCs, as depicted in Figure 1, work in the diffraction regime. For a surface GC, if the refractive index varies only in one direction, we call it a 1D-GC and light is coupled in the direction of index variation. For simplicity, we analyze the diffraction GC working principles in terms of 1D-GC, while the theories are also valid for 2D-GC if only the propagation direction works in a diffraction regime. However, if both directions work in diffraction regime, light can propagate in both directions and the 1D-GC working principles apply for both directions. For a GC with straight gratings, a spot-size converter based on tapered waveguide of hundreds of microns is required to convert the coupled fiber mode into a 500-nm waveguide mode, while a confocal grating allows compact design of GC with tens of microns in footprint.

### 2.1. Bragg Condition, Loss Channels and Fiber Tilt Angle

**Figure 2:**
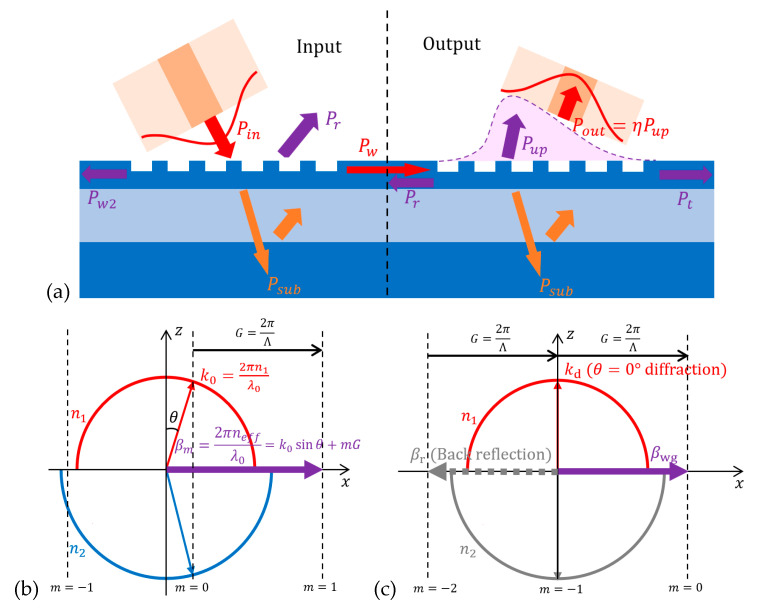
(**a**) Loss channels in input and output coupling; (**b**) wave-vector diagram for fiber-to-chip coupling; and (**c**) wave-vector diagram for chip-to-fiber perfectly vertical coupling.

The diffraction behavior for a GC, as illustrated in Figure 2a, can be described using Bragg condition (also called phase matching condition), which reveals the relationship between wave-vector $k_{0}$ of an incident light beam above chip and propagation constant **β** of the corresponding coupled light beam into the waveguide. The Bragg condition is expressed as

(1) $$k_{0}\sin\theta +mG=\beta_{m},$$

where **G** is the grating vector and *m* is the grating diffraction order. This equation is better depicted by a wave-vector diagram, as shown in Figure 2b for fiber-to-chip coupling, while a similar analysis also applies for chip-to-fiber case.

The Bragg condition only predicts which diffraction order is allowed, instead of energy distribution between different orders or diffraction efficiency. CE is usually obtained using a numerical method such as finite element time domain (FDTD) calculations [18]. To estimate the CE qualitatively, we can analyze some major power loss channels, as marked in Figure 2a. Firstly, for the input coupling case on the left side of Figure 2a, some uncoupled power will propagate downwards. Although part of that power can be reflected if the oxide thickness is properly chosen for constructive interference upwards, a considerable portion $P_{sub}$ is leaked to the substrate. Secondly, some portion $P_{r}$ is reflected to the opposite of incident direction. Thirdly, some portion $P_{w2}$ is coupled opposite to the waveguide direction, especially for perfectly vertical incidence. The final coupled power into the waveguide is then given as

(2) $$P_{w}=\eta_{CE}P_{in}=P_{in}-P_{sub}-P_{r}-P_{w2},$$

where $\eta_{CE}$ is the coupling efficiency or expressed as coupling loss in decibel (dB).

For chip-to-fiber out-coupling, as depicted on the right side of Figure 2a, a similar analysis holds and CE is usually described in terms of directionality and modal overlap. Directionality $P_{up}/P_{w}$ refers to the fraction of waveguide power that diffracts towards fiber. Modal overlap refers to the portion of upward directed power that is launched into fiber. Hence, the final coupled power into the fiber is

(3) $$P_{out}=\eta_{CE}P_{w}=\eta (P_{w}-P_{t}-P_{r}),$$

where the overlap integral between upward directed mode and fiber mode is expressed as

(4) $$\eta =\frac{\int F(x)G(x)dx}{\int F^{2}(x)dx\int G^{2}(x)dx}.$$

Although the Bragg condition allows θ=0 for perfectly vertical coupling, a fiber tilt angle is usually adopted. For chip-to-fiber case, this is explained by the strong backwards reflection due to second-order diffraction shown as $\beta_{r}$ in the wave-vector diagram (Figure 2c). The second-order reflection greatly reduces the CE for a perfectly vertical GC. For fiber-to-chip case, grating symmetry leads to bi-direction propagation of coupled light, for diffraction order m=1 and m=−1 . However, attachment of fiber without tilt angle is preferred in packaging. Therefore, many studies focus on improving the performance of perfectly vertical GC [19,20,21,22,23,24,25,26,27,28]. For the bi-directional propagation nature of perfectly vertical incidence, some designs adopt bi-directional coupling to two waveguides [25,27] while others add reflective structure to enhance coupling for only one direction [21,28]. For chip-to-fiber coupling, certain structures with reduced effective index can be placed between grating and waveguide to reduce back reflection [25,29]. Since perfectly vertical GCs have rather low CE, it is even more important for these perfectly vertical designs to adopt directionality [22,24] and modal overlap [20,25] enhancement. We discuss them in Section 3 since it also applies for GC with tilted fiber.

### 2.2. Subwavelength Grating and Effective Index Medium Theory

**Figure 3:**
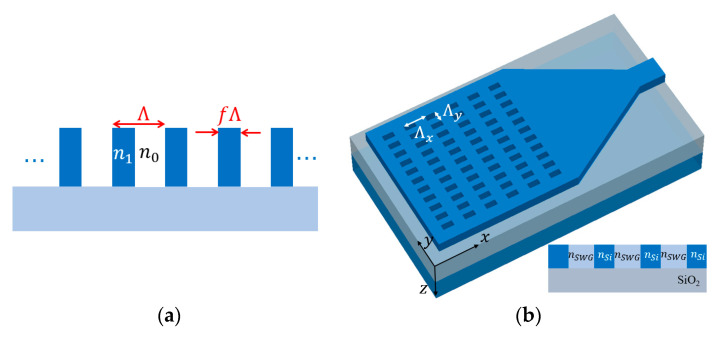
(**a**) Schematic of planar waveguide grating; and (**b**) Illustration of a subwavelength grating coupler (SWGC) (inset: its 2D equivalent index model).

Subwavelength grating (SWG) refers to grating with period small enough to suppress diffraction effects [16,17]. Under certain conditions, it behaves as a homogeneous medium and has found wide range of applications in silicon photonics in the last decade. Consider a planar waveguide grating structure with grating material index $n_{1}$ , gap material index $n_{0}$ , period Λ and fill factor f , as marked in Figure 3a. This grating structure can be modeled as an equivalent homogeneous material under two conditions. Firstly, the structure has a thickness large enough so that evanescent modes cannot tunnel through the grating. Secondly, the grating has a period small enough ( Λ≪λ ) so that diffraction is suppressed. The approximated refractive index of this equivalent medium is given by Rytov’s expression [30] as

(5) $$n_{TE}^{2}=fn_{1}^{2}+(1-f)n_{0}^{2},$$

and

(6) $$n_{TM}^{-2}=fn_{1}^{-2}+(1-f)n_{0}^{-2}.$$

SWGs allow effective index engineering in a straightforward way. By alternating SWG structure and unetched silicon in x-direction, as depicted in Figure 3b, subwavelength grating coupler (SWGC) with single patterning and single full etch step is enabled [31,32,33,34,35,36], thus fine tuning of etch depth to control effective index is avoided. By alternating subwavelength engineered structures with different effective index, apodized GC [32,34] and broadband GC [36] is achieved. SWGs are also utilized in the designs of fiber edge couplers [37,38], ring resonators [39] and multimode interference (MMI) couplers [40].

### 2.3. Transition Taper and Focusing Grating Coupler

**Figure 4:**
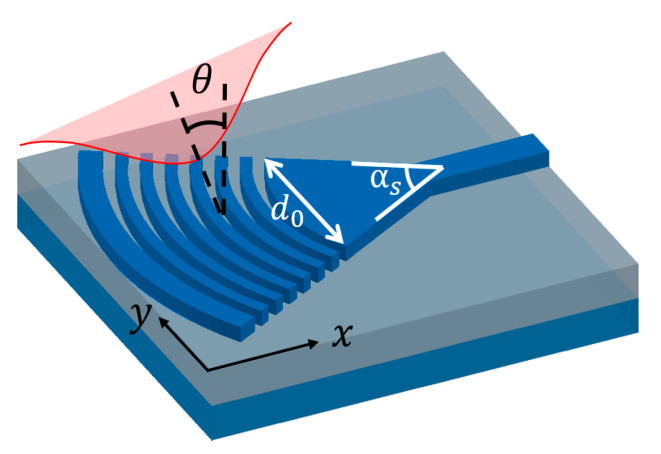
Schematic diagram of a focusing GC with curved grating lines.

Since GC interfaces the fiber mode directly, the grating width at y-direction is usually comparable to the MFD of around 10 μm. The transition of light to single-mode waveguide with 400–500 nm width is usually conducted by a tapered waveguide. If propagating mode only changes its size and shape in this transition, without radiating outside waveguide or converting to other higher-order waveguide mode, then energy is conserved. In this case, we consider it as an adiabatic transition. For a linear tapered waveguide that allows adiabatic transition, its geometric parameters must satisfy [41]

(7) $$\theta_{taper}<\frac{\lambda }{2Wn_{eff}},$$

where $\theta_{taper}$ is the taper angle, W is the varying waveguide width and $n_{eff}$ is the corresponding mode effective index, as marked in Figure 1. To ensure high CE, GC follows an adiabatic transition waveguide, usually of 300–500 μm in length, which occupies much space on chip. To reduce footprint, a focusing design of GC is usually adopted, as illustrated in Figure 4. In this design, the grating lines are curved to [42]

(8) $$m\lambda =n_{eff}\sqrt{x^{2}+y^{2}}-xn_{0}\sin\theta ,$$

where origin is the focal point, *m* is an integer for each grating line, θ is the fiber tilt angle, n_0_ is the cladding refractive index and $n_{eff}$ is the GC effective refractive index. The curved grating lines form ellipses with common focal points. In this way, the coupled wavefront will be curved and light from fiber will be focused as it propagates, thus eliminating the need for full-length adiabatic taper. For a focusing GC with grating lines curved as in Equation (8), light diffracted from different positions is expected to interfere constructively at the focal point. However, since the effective refractive index $n_{eff}$ may not be evaluated precisely for etched gratings, it is recommended to perform a sweep on the radius of curvature in simulation. For an optimum design, the CE verified by 3D-FDTD simulation can be nearly identical to its straight-line counterpart. After setting the grating curve, the size of a focusing GC is determined by section angle $\alpha_{s}$ . Since the first grating line span on y-axis $d_{0}$ should be comparable to MFD, footprint for a focusing GC can be further reduced by choosing a larger sector angle $\alpha_{s}$ . However, this may increase roughness on the curves as they are represented by polygons in lithography, which induces extra scattering loss. Thus, a balance is required considering fabrication limitations and also the footprint.

## 3. Coupling Efficiency Enhancement

As illustrated in Figure 2a, CE of GC can be improved by enhancing directionality (or reducing back-reflection and substrate loss) and increasing modal overlap. We first discuss adding overlay on grating or reflector below grating to increase CE, and then review structural modifications of SOI grating without adding fabrication steps other than etch. We present a comparison of the different coupling efficiency enhancement schemes of GCs reviewed in this section in Table 1.

### 3.1. Additional Fabrication Techniques for Directionality

#### 3.1.1. Poly-Silicon Overlay

**Figure 5:**
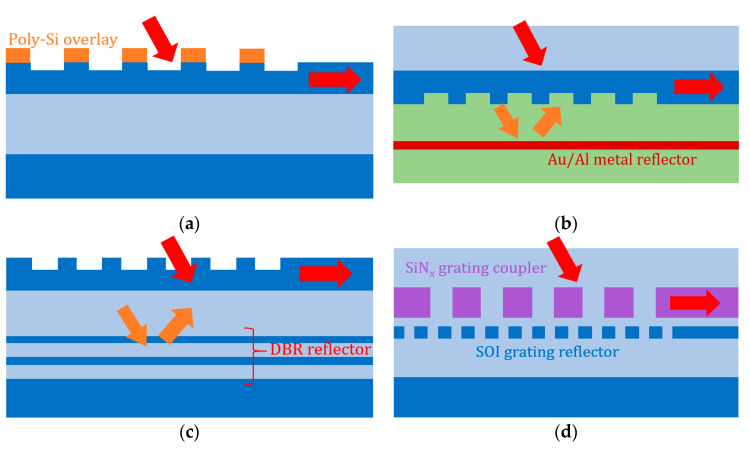
Additional fabrication techniques to enhance GC directionality: (**a**) GC with poly-silicon overlay; (**b**) GC with metal reflector, fabricated by flip-chip and benzo-cyclobutene-bond; (**c**) GC with distributed Bragg reflector; and (**d**) GC with silicon grating reflector on silicon-nitride-on-silicon platform.

Diffraction grating on SOI has limited directionality, due to the similar refractive index of superstrate (SiO_2_ or air) and buried oxide below grating (SiO_2_). One widely investigated approach to enhance directionality is by modifying the grating structure so that different scattering centers have a constructive interference towards superstrate and a destructive interference downwards to the substrate [43,50]. By adding a poly-silicon layer deposition prior to grating etching as in Figure 5a, a highly directional GC can be obtained with the depth of each grating teeth higher than the thickness of SOI. For upwards propagation of radiated beam, the additional poly-silicon thickness provides additional phase difference among scattering centers. Constructive interference towards fiber can be achieved by properly choosing the poly-silicon thickness and directionality can be maximized in this way.

#### 3.1.2. Bottom Reflector

Apart from intrinsic property of grating structure, directionality can also be increased by “recycling” downwards radiated power. When light is diffracted towards the substrate, a part of the power reflects towards grating at the oxide/substrate interface. This reflection can be enhanced by optimizing the oxide layer thickness to achieve constructive interference, but it requires custom SOI. A straightforward approach to maximize this reflection is to insert a reflector, such as metal with close to 100% reflectivity. Figure 5b is an example of SOI flip-chip and BCB-bond with metal reflector to realize high efficiency GC [44,51,52,53,54]. With metal mirror, many GCs with sub-decibel CE is achieved. However, this approach requires bonding nonstandard in CMOS-compatible process. Another similar approach is to fabricate a distributed Bragg reflector (DBR) such as in Figure 5c, which is also complex for device processing [45,55]. To our best knowledge, thus far, all high-performance GCs with sub-decibel measured CE adopt bottom reflector. Therefore, directionality enhancement using bottom reflector is still worth exploring, even though complex fabrication techniques are involved. In recent years, multi-layer silicon-nitride-on-silicon platform has developed, which provides an elegant approach to realize bottom reflector: grating reflector on silicon layer [46,56,57], as shown in Figure 5d.

### 3.2. Grating Structural Innovation

#### 3.2.1. Apodized Grating Coupler to Increase Modal Overlap

As depicted in Figure 2b, one major GC loss is due to the modal mismatch between Gaussian fiber mode and grating diffraction profile. Since the grating diffraction profile at y-direction is Gaussian-like, we only discuss its variation in x-direction. For a uniform GC, each diffraction unit has the same ability to diffract light (referred to as coupling strength α). Thus, the grating diffracted field G(x) is exponentially decreased, which is expressed as

(9) $$G(x)=G(0)\exp[-\alpha_{0}{\left(x-x_{0}\right)}^{2}].$$

**Figure 6:**
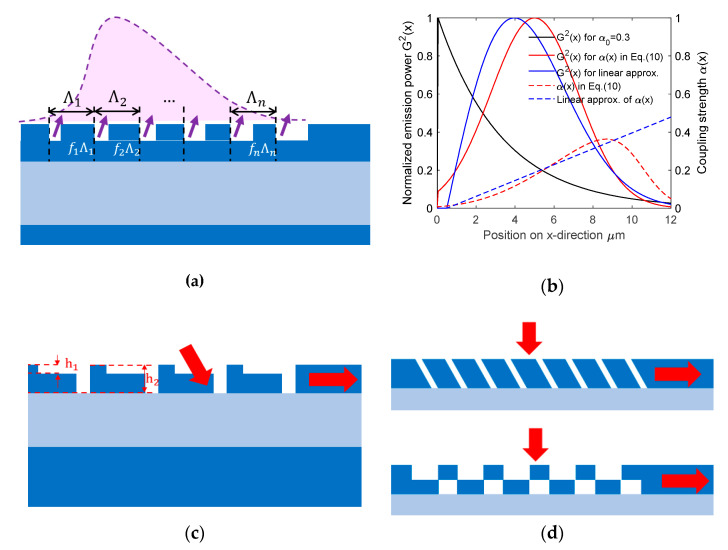
Structural innovations on GC: (**a**) apodized GC with varied duty cycle $f_{n}$ or period $\Lambda_{n}$ for different grating teeth; (**b**) theoretical calculation of normalized output power distribution (solid) and coupling strength (dashed) for ideally (red) and linearly (blue) apodized GCs. (**c**) GC with double etch steps; and (**d**) slanted GC (upper) and dual-layer GC (lower).

For G(x) to match the fiber Gaussian field distribution, we can vary the coupling strength along x-direction to get an “apodized” GC [55,58]. Gaussian output beam can be obtained by replacing $\alpha_{0}$ with

(10) $$\alpha (x)=\frac{F^{2}(x)}{2*[1-\int_{0}^{x}F^{2}(t)dt]},$$

where F(x) is the normalized fiber Gaussian mode profile. We plot the calculated α(x) and corresponding output power profile in Figure 6a. The figure also shows that linear variation is a good approximation, which is adopted by many reported apodized grating coupler designs. For 1D GC, varying coupling strength α(x) is usually performed by varying duty cycle (or fill factor) $f_{n}-f_{n-1}=\Delta f$ for etch grating trench along x-direction [47,55], by varying grating period [20,59] or the two combined [59], as shown in Figure 6b. For 2D SWGC, by varying the fill factor for SWGs, the effective index is changed along x-direction to achieve apodization [31,60]. Etch depth apodization is also investigated, where gradual change of etch depth is created using lag effect in ICP-RIE etching [61].

#### 3.2.2. Complexity in Z-Direction to Increase Directionality

For poly-silicon overlay GC, the enhanced directionality is attributed to the increased grating structure asymmetry in z-direction. There are many research works focused on asymmetry within the silicon waveguide layer. The most popular approach is to introduce multiple (usually two dues to the difficulty in alignment) etch depths within top silicon layer [29,48,62,63,64,65,66,67], as shown in Figure 6c. Several dual-etch GCs are proposed with high CE. Although many silicon photonics multi-project wafer (MPW) service provides multiple etch depth, the difficulty in realization of dual-etch GC lies in the requirement of alignment between patterns with different etch depth, which is usually tens of nanometers. Other complexity schemes including slanted grating [49,68] and dual-layer grating [69,70,71] in Figure 6d are also investigated, but with little feasibility for massive manufacturing. Slanted grating, for instance, requires focused ion beam (FIB) for fabrication.

## 4. Polarization and Wavelength Diversity

### 4.1. Polarization Diversity

The output beam polarization from SMF is constantly changing due to polarization mode dispersion. However, GCs are polarization selective due to the strong birefringence of SOI. The polarization uncertainty from fiber and sensitivity from GC influence the power of light coupled to on-chip waveguides. Therefore, it is important to introduce polarization diversity for receiver GC, while for chip-to-fiber emitter GC single polarization should be enough. Table 2 provides a comparison of different polarization diverse GCs reviewed in this section.

#### 4.1.1. Polarization Splitting Grating Coupler (PSGCs)

**Figure 7:**
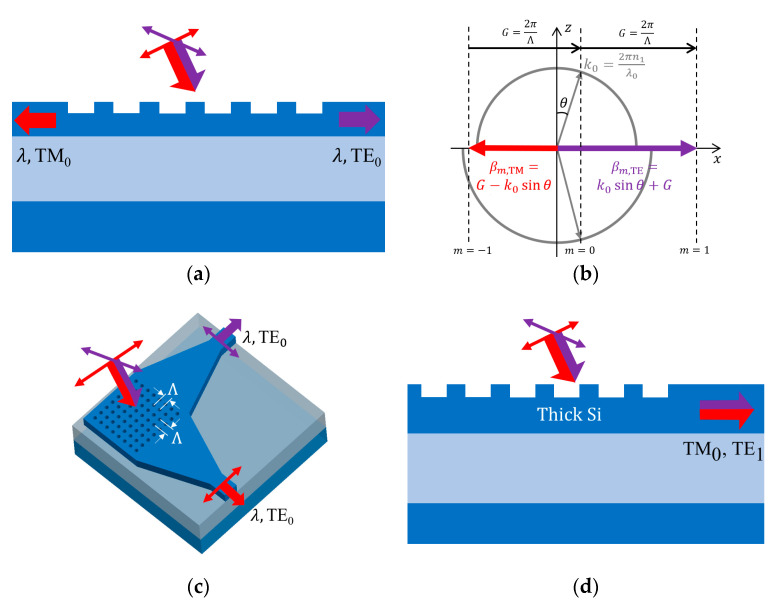
GCs with polarization diversity: (**a**) 1D polarization splitting grating coupler (PSGC) with traverse electric (TE)/ traverse magnetic (TM) polarizations coupled to TE_0_/TM_0_ waveguide modes in contrary directions; (**b**) wave-vector diagram for 1D PSGC; (**c**) 2D PSGC with TE/TM polarizations coupled to TE_0_ waveguide modes in orthogonal directions; and (**d**) thick silicon-on-insulator GC for polarization-insensitive coupling.

PSGCs can be designed as both 1D GC [53,72] and 2D GC [54,67,73,74,75,76,77,78,84]; in both cases, two polarizations are coupled into waveguides at different directions. For 1D PSGC, TE and TM polarizations are coupled in contrary directions using diffraction order m=±1 in Equation (1). Figure 7a is an example of 1D PSGC; its k-vector diagram is shown in Figure 7b. More research works are focused on 2D PSGC. It functions as two orthogonally arranged 1D GCs combined, each coupling light of its corresponding polarization into TE mode in the waveguide it connects, as illustrated in Figure 7c. Fiber incidence plane is rotated by 45° for symmetry, while remaining tilted off-normal for better CE. Incoming beam from fiber with arbitrary polarization state can be decomposed into two orthogonal linear components that can be converted to TE modes in two waveguides. Even when polarization state varies, the sum of total power coupled to the two waveguides remains almost unchanged. Therefore, the 2D PSGC is polarization insensitive. Due to the fiber tilt angle, refraction occurs in SOI and coupled mode propagates with a small angle offset from the two orthogonal directions, typically 3–4° depending on the tilt angle.

#### 4.1.2. Unidirectional Polarization Insensitive Grating Coupler

Some GC designs overcome the polarization dependence without splitting them up. One approach is to eliminate the birefringence by engineering subwavelength effective index medium. For example, in [81], both first and second orders of effective index medium theory equations are investigated in the design, so that the effective indices are made similar for both TE and TM modes to support polarization insensitive operation. It is also possible to couple TE and TM polarization using different grating diffraction order, or coupling into different order of waveguide mode, as shown in Figure 7d, while these methods usually adopt thick SOI to reduce birefringence [82,85,86]. Structural innovations are also investigated for polarization diverse coupling in 1D GC, including dual-etch grating [79], intersection and union of TE and TM GCs [83], as well as non-uniform GCs using inverse design [87,88] that we describe in detail in Section 5.2.

### 4.2. Wavelength Diversity

Bragg condition suggests that GC is highly wavelength sensitive, since radiation angle is wavelength dependent while the optical fiber only accepts light with a small range of diffraction angles. The 1-dB BW of GC is usually less than 50 nm, which limits its broadband applications. Depending on operation requirements, there are basically two solutions: GC with BW high enough to cover the required wavelength range and GC with separated multiple operation bands. We discuss the operation principles and examples of these two types of GCs in this section.

#### 4.2.1. Broadband Grating Coupler

The 1-dB coupling BW for a GC is analyzed in many works [36,89,90,91]. Basically, these analyses hold similar conclusions that the BW is related with the fiber numerical aperture and the grating dispersion. In other words, for different wavelengths, the diffraction angle is different while the fiber remains fixed. We take a simplified derivation in [89] as an example. Consider a GC with designed peak coupling wavelength $\lambda_{0}$ and corresponding diffraction angle $\theta_{0}$ . For a wavelength that deviates from central, assuming grating effective index $n_{eff}$ unchanged, the relationship between diffraction angle dispersion Δθ and wavelength deviation Δλ can be derived by substituting $\lambda =\lambda_{0}+\Delta \lambda$ and $\theta =\theta_{0}+\Delta \theta$ into Equation (1), obtaining:

(11) $$\frac{\Delta \lambda }{\Delta \theta }=-\frac{\lambda_{0}n_{0}\cos\theta_{0}}{n_{eff}-n_{0}\sin\theta_{0}}.$$

For a fiber with numerical aperture NA, the allowed Δθ before CE drops 1 dB is proportional to NA, assuming grating emission profile and fiber mode are both Gaussian-shaped. Therefore, the 1-dB BW for a GC is given by

(12) $$\Delta \lambda_{1dB}=C\cdot NA\cdot \frac{\lambda_{0}n_{0}\cos\theta_{0}}{n_{eff}-n_{0}\sin\theta_{0}}$$

where C is a constant. This equation indicates that decreasing $n_{eff}$ or increasing $\theta_{0}$ can increase the 1-dB BW for a GC. A more comprehensive derivation considering the effective refractive index dispersion is given in [91], which suggests that minimizing $dn_{eff}/d\lambda$ also increases BW. Following these analyses, these methods are generally adopted for a broadband GC:

- Reducing $n_{eff}$ by choosing material with lower refractive index, such as silicon nitride. In [89], a SiN GC with -4.2 dB CE and 67 nm 1-dB BW is achieved. Due to its low refractive index contrast that offers less coupling strength, the CE is usually lower than that of SOI GCs. This is improved in [46], where a bottom Si-grating reflector is added to achieve -1.3 dB CE with 80 nm 1-dB BW.
- Reducing $n_{eff}$ by engineering effective refractive index medium in SWGC. In [92], a SWGC that intends to lower $n_{eff}$ is measured with -7 dB CE and 80 nm 1-dB BW. Another SWGC design in [36] yields 5.8 dB CE and 90 nm 1-dB BW in experiment, while it is also shown using comparison that lowering $n_{eff}$ does contribute to larger BW, but CE is reduced similar to the SiN GC case.
- Allowing the optical fiber to accept more dispersed light. One way is to use optical fiber with high numerical aperture [93], as suggested in Equation (12). Another way is to insert a Si-prism between fiber and chip to compensate the angular dispersion, where 1-dB BW of 126 nm is demonstrated in [94]. Unlike changing the grating design or material directly, this method requires either nonstandard SMF or additional fabrication effort, and it also increases the requirement for alignment accuracy.

The BW for GCs is dependent on multiple parameters involving cladding refractive index, fiber NA and more importantly grating structure that influences $n_{eff}$ and $dn_{eff}/d\lambda$ . Many reported GCs claim to be “broadband” but lack detailed theoretical explanations, or the BW is analyzed by sweeping or optimizing certain parameter set that is not general for other designs to follow.

#### 4.2.2. Dual-Wavelength-Band Grating Coupler

For applications that need two distinct and well separated wavelength bands, broadband GC cannot suffice and an advisable solution is to allow dual-wavelength-band coupling on GC. One such envisioned scenario is the use of integrated transceivers for passive optical networks (PON) [95]. Typical PON systems have two wavelengths near 1310 and 1490/ 1550 nm required for upstream and downstream signals channeling, which requires the photonic integrated circuit to perform dual-wavelength-band coupling and duplexing. Other applications may include on-chip amplification by coupling pump and signal lights simultaneously and integrated Raman spectrometry [96,97].

**Figure 8:**
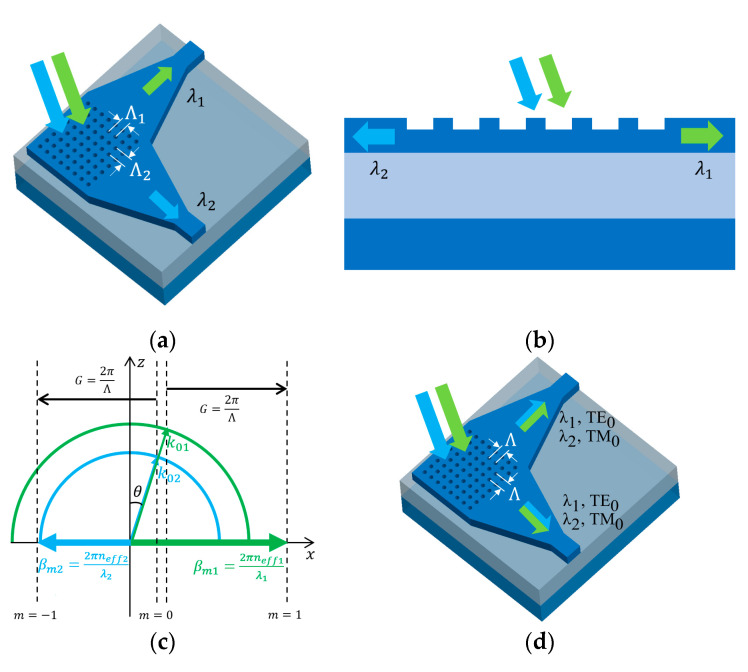
Dual-wavelength-band GCs: (**a**) two bands coupled to orthogonal waveguides; (**b**) two bands coupled to opposite waveguides; (**c**) wave-vector diagram for 1D dual-wavelength-band GC; and (**d**) 2D PSGC that supports dual wavelength bands.

Since the difference in separated wavelength bands is mainly in effective refractive index, many dual-wavelength-band mechanisms can be inspired from polarization handling. For example, one approach, as depicted in Figure 8a, is to utilize a 2D PSGC structure for different bands at the two orthogonal directions with different grating periods [98]. Similarly, a 1D PSGC structure can also be utilized for two bands coupling using diffraction order m=1 and m=−1 in the opposite direction [99], as depicted in Figure 8b,c. The effective refractive index difference for the two bands may compensate the TE/TM mode refractive index difference, so that the two bands are coupled into different modes in waveguide, such as the 2D PSGC [84] illustrated in Figure 8d. Unidirectional coupling of TE/TM mode is also investigated for two bands [100,101,102]. These dual-wavelength-band GCs are compared in Table 3.

## 5. Emerging Trends in Grating Coupler Research

### 5.1. Space Division Multiplexing Using Grating Couplers

**Figure 9:**
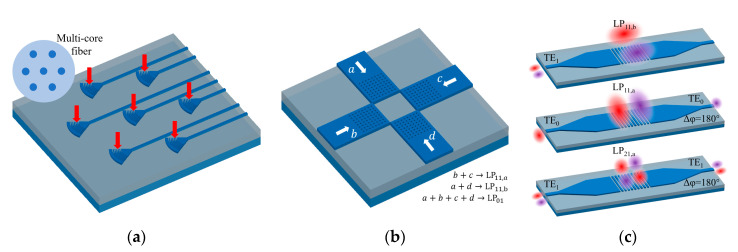
(**a**) Multi-core fiber (MCF) multiplexer based on multiple GCs [105]; (**b**) Few-mode-fiber mode multiplexer based on multiple GCs [114]; and (**c**) FMF mode multiplexer based on single GC [118].

To increase transmission capacity of systems based on silicon photonics with SMF, wavelength division multiplexing, polarization multiplexing and advanced modulation formats are all exploited. In recent years, space division multiplexing (SDM) using few-mode fibers (FMFs) [103] and multi-core fibers (MCFs) [104] attracted attention for their capability of further increasing capacity. For silicon photonics, MCF interface can be achieved using multiple grating couplers with accurate spacing to match the spacing of multiple cores [105,106]; one example is shown in Figure 9a. While spatial light modulator [107] and photonic lanterns [108] are proposed for FMF mode multiplexing, direct interface between FMF modes and waveguide modes on silicon photonics is still a challenge. In recent years, practical implementations of mode division multiplexing (MDM) on silicon photonics have already been proposed [7,109,110,111], which promote the demand for direct FMF mode-multiplexing interface on chip. To the best of our knowledge, for higher-order mode interface using GC, thus far only LP_11_ mode experimental demonstration has been reported [112,113,114,115,116,117,118,119,120]; LP_21_ mode coupling using GC is simulated in some works [118,121], but experimental implementations are still desirable. Higher-order FMF modes consist of multiple lobes in its electric field profile, while neighboring lobes have opposite phase. It is important to control their phase difference to be π , while the origin of this phase difference could come from silicon thermal phase tuning for different lobes, π -phase difference in TE_1_ waveguide mode, as well as intrinsic phase displacement inside the gratings. We categorize different published works of FMF mode coupling GCs by these schemes, and we review them one by one in the following text.

The first approach for FMF mode multiplexing is to use multiple independent GCs [112,113,114,115,116]. The inputs of these independent GCs are all fundamental TE mode, usually with phase controlled by thermal phase shifters. Obviously, this approach requires extra power consumption for operation and current experimental demonstrations lack in CEs. For example, in [114], fundamental waveguide mode inputs are diffracted by four carefully located small GCs, as depicted in Figure 9b. If there are two inputs, the diffracted beams will form the two lobes for a LP_11_ mode. Phase shifter tunes the phase of the two beams to be exactly opposite, in order to ensure the quality of the excited fiber mode. By switching input ports, LP_01_, LP_11a_ and LP_11b_ modes with different polarizations are demonstrated. Total insertion loss of 23 dB from SMF input to FMF output and mode dependent loss of 2 dB is measured, while insertion loss is reduced to 10.6 dB by adding a bottom metal reflector in [115]. Configurations using three [112] and five [113] GCs are also reported.

The second approach is to excite higher-order FMF modes using TE_1_ waveguide mode [117,118,119], not necessarily with thermal phase shifters. We take the GC illustrated in Figure 9c as an example [118]. Unlike narrow waveguides, the effective refractive index difference between TE_0_ and TE_1_ mode is small for GCs since they are wide enough. Thus, the GC used for TE_0_ mode out-coupling also works for TE_1_ mode, and the launched beam resembles LP_11_ mode due to the field distribution of TE_1_ waveguide mode. To improve the performance and allowing LP_11a_ mode, this work also has two inputs at two different ends of the GCs, while this additional enhancement does require phase shifter. In [119], similar operation without inputs from opposing ends and phase shifters is investigated. Another demonstration in [116] does not interface TE_1_ mode directly with GC, a Y-junction splits it into two TE_0_ modes that maintain the opposite phase.

Non-uniform or segmented GCs with built-in phase difference are also investigated for FMF mode coupling [120,121]. In [121], π phase difference is introduced at the position corresponding to the separation of lobes in the output beam. For LP_11_ mode, π-shift is applied in x-direction or y-direction of the grating. For LP_21_ mode, π-shift is applied in both directions to form the diffracted beam with four lobes. Since it only couples one specific FMF mode, multiplexing is not achieved.

### 5.2. Objective-First Design for Grating Couplers

The development of silicon photonic devices has long relied on intuition-based approaches, by applying specific features with known physical effects, and tuning small sets of parameters to utilized the effects to meet the demand in applications [122]. In recent years, an objective-driven approach named “inverse design” has been explored in silicon photonic devices mainly due to two reasons. This first reason is to break fundamental limits of previous intuition-based device design methodology. One representative is the device footprint. Many ultra-compact devices based on inverse design are proposed, with comparable performance to traditional devices [123,124,125,126]. This is partly due to the increased design complexity offered in certain confined design area, which is not fully explored in intuition-based design procedures. The second reason is to save research effort. The inverse design approach is similar to optimizing a black box to achieve certain performance metrics, while the iterative optimization and performance verification are fully automated using predefined computer algorithms. Inverse design on GC has already been reported in several research works [70,71,88,127,128,129,130,131]. Most of these works offer an automated design approach for non-uniform 1D GC devices with design goals already addressed in previous works reviewed above, such as increased CE, broadband operation and polarization independence. In addition, this approach also produces unimagined topological structures that achieved compact coupler without transition taper and high-order fiber modes coupling and splitting [128].

However, inverse design also comes with many drawbacks. First, the extra complexity that inverse design explores may not be fully satisfied using current fabrication technologies. The reported inverse designed GCs usually involve high-resolution patterns that only e-beam lithography can produce. Second, this computer-driven design procedure has high requirement on computing power for the iterative optimization, especially when reliable electromagnetic numerical simulation such as 3D-FDTD is adopted. Therefore, many inverse design research works focus on component “compactness” with small simulation area requirement that can be evaluated and optimized fast. GCs are not suitable for such compactness since it must interface fiber mode with ~10 μm MFD. In the future, efforts on efficient optimization algorithm as well as fast electromagnetic simulation may accelerate the adoption of objective-first design.

### 5.3. Grating Couplers on Different Material Platforms

#### 5.3.1. Multi-Layer Silicon Nitride Grating Coupler

Silicon nitride (SiN) is a promising candidate for silicon photonics due to its many advantages over silicon [132,133,134]. First, it has low propagation loss, especially in O-band. Waveguide propagation loss is largely contributed by scattering loss due to nanometer-scale sidewall roughness. The relatively low refractive index contrast with SiO_2_ brings less light confinement, so that SiN waveguides are usually larger than Si waveguides in size. Consequently, they are less sensitive to the nanometer-scale roughness than Si waveguides. The second advantage is its manufacturing flexibility. SiN can be deposited using low-pressure chemical vapor deposition (LPCVD) at high temperature or plasma-enhanced chemical vapor deposition (PECVD) at low temperature, compatible with current silicon photonics manufacturing process. Multi-layer integration is already demonstrated using SiN, which attracted great interest in recent years. In addition, there are some other advantages for SiN, including low nonlinearity which can support high power, wide transparency range that extends to visible, as well as low thermal sensitivity. These above-mentioned advantages make SiN particularly suitable for passive silicon photonics devices.

**Figure 10:**
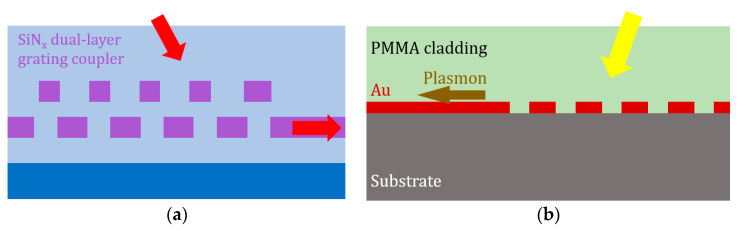
(**a**) Dual-layer SiN-on-SiN GC; and (**b**) metal GC that excites surface plasmons.

In terms of fiber coupling, while the SiN platform is demonstrated to be advantageous for edge couplers [133], the characteristics of SiN have both advantage and disadvantage for vertical GCs. On the one hand, the low refractive index provides low coupling strength. Before multi-layer SiN platform was developed, many GC research works on SiN reported relatively low CE, typically with insertion loss larger than 4 dB [89,135]. On the other hand, the multi-layer capability adds new degree of freedom to GC design. Firstly, complexity in z-direction shown previously is easily achieved on multi-layer SiN. Increased CE and polarization independence are demonstrated using dual-layer SiN-on-SiN platform in Figure 10a, with grating patterns differ in the two layers [133,136,137]. Secondly, GC with bottom reflector is more easily combined with SiN platform than SOI, since SiN layers can be directly deposited above the reflective structures. One example is the Si-grating based reflector reviewed previously [46,56,57]. Its deposition flexibility also facilitates the adoption of metal [51] or DBR [45] reflector that is difficult to combine with single-crystalline silicon waveguide devices.

#### 5.3.2. Plasmonic Grating Coupler

Surface plasmon polaritons (SPPs) are electromagnetic surface waves propagating at the interface of a dielectric and a metal. Plasmonic waveguides enable surface plasmon modes with extremely high electric field confinement, thus greatly increasing the compactness for photonic integrated circuits. They can also be utilized for biosensing with biomaterial binds to the surface and changes the effective refractive index of the plasmonic waveguides. Schemes for conversion of beam from optical fiber or free-space into SPPs include prisms, edge couplers, antenna structures and GCs [138,139,140,141]. GC is advantageous in terms of easy fabrication and simple structure. However, high-resolution lithography is required since plasmonic GCs are much more compact than photonic ones. In contrast to photonic GC, the plasmonic GC in Figure 10b is based on periodic metal grating patterns rather than dielectric, and the diffraction mechanism does not come from refractive index fluctuation but from strong resonant scattering at the metallic grooves. Phase matching condition still applies, and many photonic GC design principles such as apodization and focusing grating strips are also adopted in various research works on plasmonic GCs. In [139], an apodized plasmonic GC on dielectric-loaded surface plasmon polariton waveguide is experimentally measured with 2.9 dB peak CE. GCs on other material platforms such as lithium niobate on insulator [142,143] have also been investigated.

## 6. Practical Issues for Grating Couplers

### 6.1. Wafer-Level Testing and Packaging

One key advantage of grating coupler is that wafer-level optical testing is enabled, which allows manufacturers to determine the quality of dies on wafer and saves further cost for packaging and final quality control of bad dies. Unlike electrical measurement using DC probes, optical testing on GCs relies on fiber probes and come with problems including polarization control, ease damage of probe if hit by chip, as well as stringent positional requirement. Currently, positioning of the probe is usually done by active alignment, where laser source and detector is tuned on during position switching for maximum power coupling. Optical measurement can be done by either wavelength swept laser source and photodiode, or broadband source such as supercontinuum and optical spectrum analyzer. In this process, polarization control is crucial, due to the large polarization dependence of silicon photonic devices and the uncertainty of polarization caused by mechanical stress or temperature induced fiber birefringence. While a polarization controller for all polarization states can be adopted, calibration of polarization needs considerable points of power measurement that takes time. Muller-matrix analysis is available to determine maximum coupling power and PDL by quick measurements of only four fixed polarization state [144,145].

**Figure 11:**
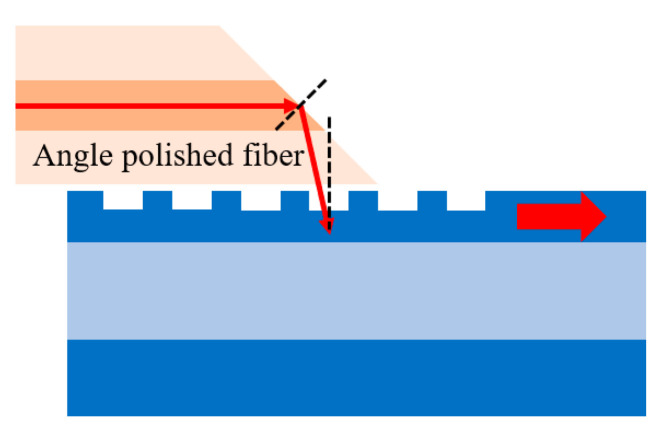
Lateral coupling by angle polished fiber.

Optical packaging is also an important step in silicon photonics for commercial mass-production, since its cost is considerable in the overall module fabrication. GC is preferred in packaging since it provides higher alignment tolerance and facet polishing is not required. There are mainly two approaches adopted for GC-based packaging. Since for grating coupling the light incidence is vertical, a straightforward way is to “pigtail” the fibers vertically on chip [146]. One example of this approach is the “g-Pack” standard offered by commercial service ePIXpack, where standardized fiber array is mounted with a glass block polished to provide 8° tilt angle on a v-groove carrier [147]. Hermetic sealing not offered by “g-Pack” is also investigated in to other works such as by quad flat no-lead package widely used for microelectronic device packaging [148,149]. This approach, however, occupies much space for further component integration and offers poor mechanical strength. Therefore, the lateral coupling by angle polished fiber in Figure 11 is pursued [150,151,152], and its mechanical stability can be offered by three-point contact from etched v-grooves plate and top planer glass lid [153].

### 6.2. Applications

#### 6.2.1. Photonics Integrated Circuits (PICs)

This article is mainly organized to discuss the design of GCs to serve as a fiber I/O for PICs, mostly applied in communications. Increasing CE is important to save power budget. Increasing wavelength diversity, either by BW enhancement or multi-wavelength-band operation according to practical requirements, can contribute to more channels in WDM. Allowing polarization diversity reduces PDL in receivers side. Exploring coupling to MCF or higher-order mode multiplexing in FMF also increases the channels ready for use. All these goals for GC designs reviewed by this article contribute to higher data capacity. Besides fiber coupling, GC can also apply in other coupling issues in photonic integration. The first is on-chip light source coupling to waveguide. For example, in [154], an InP distributed feedback (DFB) laser is coupled to SiN waveguide via two GCs on InP and SiN respectively. This coupling solution allows insertion of an isolator between the GC on laser and the GC on Si chip, while the two GCs already function as polarizers for the isolator. The second is inter-layer coupling in multi-layer PICs. Materials such as SiN allow multi-layer interconnect to increase complexity and integration density, while interlayer light-guiding using GCs is also investigated [155,156]. Compared with other interlayer coupling scheme [134], GC is advantageous in that large separation between layers is allowed.

#### 6.2.2. Biomedical Sensing

As mentioned above, GC is used to excite surface plasmons for biosensing. Compared with other methods to excite surface plasmons, GC is advantageous in that uncoupled light propagates orthogonally to the sensing wave, providing higher signal-noise ratio. In addition, GC itself is also a good candidate for biomedical sensing for its simplicity in principle and ease in fabrication. The dielectric grating evanescent wave is sensitive to refractive index variation caused by proteins, cells and drug reaction, which changes the diffraction angle or peak wavelength in the Bragg condition in Equation (1). Many GC biosensors are made by injecting light beam in different incident angles to the GC and detecting the coupled power to waveguide [157,158].

#### 6.2.3. LiDAR

Beam-steering devices play an important role in LiDAR systems that are widely used in autonomous vehicle, robots and mapping. At present, on-chip beam-steering LiDAR using technologies such as optical phased array (OPA) has attracted great attention due to its prospect of drastic cost reduction in manufacturing [159,160,161]. In OPAs, beam-steering is achieved through adjusting the phase difference between on-chip GC emitters for constructive interference at desired directions. One important challenge for chip-based LiDAR is the high loss on MMI-trees and out-coupling; GCs with high CE will help improve chip-based LiDAR performance. In addition, the far-field beam shape for OPA can be optimized at near-field using apodized GC emitters, similar to what is reviewed above in fiber coupling.

## 7. Conclusions

Efficient coupling is an important aspect of silicon photonics. Among the two most popular coupling schemes, GCs are preferred for their flexibility, easy fabrication and testing, but are disadvantageous in coupling loss, polarization and wavelength diversity that edge couplers are advantageous in. In the past decades, research works have proposed various methods of performance enhancement to tackle these drawbacks, which we review separately by classifications, explanations and comments. CE for a GC can be enhanced by overlay or bottom reflector, while structural innovation also provides increased efficiency without complex fabrication processes. Polarization diversity is important at receiver side, which can be handled by improved designs such as PSGCs; limited BW can be addressed using methods derived from BW analysis, while dual-wavelength-band GCs provide another possibility for wavelength diversity. Apart from these performance enhancements, recent research efforts also explore SDM using GCs to increase communication channels and computer-aided design for GCs, as well as new characteristics of GCs on other material platforms for silicon photonics. At last, we provide extra knowledge in testing, packaging and applications of GCs. GCs are not only used to couple light between fiber and chip, they also couple between different layers integrated on chip, and between on-chip light source and waveguides. GCs implemented for other applications including biomedical sensing and LiDAR are also briefly reviewed.

## Figures and Tables

**Table 1:** Comparison between some grating couplers (GCs) with features reviewed in Section 3.

Ref.	Section	Features Description	Peak CE (dB)	BW (nm, Exp.^1^)	Comments
[43]	3.1.1	SOI GC with overlay	Sim.^1^: −1.0 Exp.^1^: −1.6	44 (1 dB)	Polysilicon overlay enhances the upwards directionality for GCs.
[44]	3.1.2	SOI GC with Au bottom reflector	Sim.: −1.1 Exp.: −1.6	45 (1 dB)	Metal reflector enhances directionality by “recycling” substrate leakage, but requires complex fabrication processes.
[45]	3.1.2	SiN GC with DBR reflector	Sim.: −2.3 Exp.: −2.5	53 (1 dB)	DBR is another type of reflector that deposited layer by layer, compatible with SiN fabrication process. Reflectivity restricted by the number of layers.
[46]	3.1.2	SiN GC with SOI grating reflector	Sim.: −1.0 Exp.: −1.3	80 (1 dB)	SOI grating can also be an efficient bottom reflector, it is compatible with SiN multilayer integration.
[47]	3.2.1	SOI GC, duty-cycle apodized	Exp.: −3.1	41 (1 dB)	The diffraction field profile of apodized GC has a good overlap with fiber mode, which enhances CE without additional processes.
[34]	3.2.1	SOI SWGC, effective index apodized	Sim.: −2.0 Exp.: −2.2	64 (3 dB)	Apart from duty-cycle and period, SWG effective index can also be apodized for SWGC.
[48]	3.2.2	SOI SWGC, dual-etch	Sim.: −1.1 Exp.: −1.3	52 (3 dB)	Multiple etch depths is another option to enhance directionality without introducing overlay or reflector.
[49]	3.2.2	Slanted GC	Sim.: −1.9 Exp.: −3.4	80 (3 dB)	Slanted GC is one option to enhance directionality, but fabrication requires FIB, and results differs from simulation.

^1^ Sim. refers to simulation results, Exp. refers to experimental results.

**Table 2:** Comparison of polarization diversity in GCs reviewed in Section 4.1.

Ref.	Year	Feature Description	Peak CE (dB, Sim.)	Peak CE (dB, Exp.)	PDL (dB, Exp.)	Operation Band
[72]	2009	1D PSGC	−3	/	/	C
[53]	2013	1D PSGC with Al bottom reflector	−1.1	−2.4	/	C
[73]	2003	2D PSGC first proposal	/	−7	/	C
[74]	2014	2D PSGC on double-SOI substrate	/	−2	/	S
[27]	2015	Perfectly vertical 2D four-port PSGC, bi-direction propagation	/	−4.8	/	C
[54]	2018	2D PSGC with gold bottom reflector	−1.4	−1.8	1	C
[67]	2019	Dual-etch 2D PSGC	−2.4	−2.6	0.8	C
[75]	2015	Four-port fully-etched 2D PSGC with unique grating cell	−5.8	−6	0.2	C
[76]	2016	2D PSGC with unique grating cell	−4	−4.4	0.25	C
[77]	2018	2D PSGC with unique grating cell	−3.4	−4.2	0.2	C
[78]	2020	2D PSGC with unique grating cell and gold bottom reflector	−1.7	−2.4	0.2	O
[79]	2010	T-shaped polarization-insensitive GC	−2.4	/	/	C
[80,81]	2011& 2014	Polarization-insensitive SWGC on 340 nm SOI with DBR reflector	−2.5	TM: −3.2 TE: −4.3	1.1	C
[82]	2012	Polarization-insensitive GC on 1.5 μm SOI, 0.75 μm-thick waveguide	−2.8	/	/	C
[83]	2016	Non-uniform GC by union/intersection of TE/TM GC	TE: −6.9 TM: −7.1	TE: −7.9 TM: −7.4	0.5	C

**Table 3:** Comparison of dual-wavelength-band GCs reviewed in Section 4.2.2.

Ref.	Year	Feature Description	Peak Wavelength (nm)	Peak CE (dB, Sim.)	Peak CE (dB, Exp.)
[99]	2007	Coupling to contrary directions, with overlay	1310 1490	−2.6 −2.5	/
[98]	2011	Coupling to orthogonal directions, like 2D PSGCs	1480 1530	/	−6.5 −6
[84]	2013	2D PSGC for both bands, O/C-band couple to TM/TE waveguide modes	1300 (to TM mode) 1550 (to TE mode)	/	−8.2 (to TM mode) −7.1 (to TE mode)
[100]	2018	Shallow etched SiN GC	1290 1550	−4 −4.7	−8.2 −7.3
[101]	2018	SWGC on suspended-membrane waveguide	1486 1594	−3.3 −3.7	−7.4 −7.0
[102]	2019	SWGC on suspended-membrane waveguide	1560 2255	−3.4 −1.7	−6.9 −5.9
