# Correction: Cheng et al. Grating Couplers on Silicon Photonics: Design Principles, Emerging Trends and Practical Issues. *Micromachines* 2020, *11*, 666

**DOI:** 10.3390/mi13040606

**Published:** 2022-04-13

**Authors:** Lirong Cheng, Simei Mao, Zhi Li, Yaqi Han, H. Y. Fu

**Affiliations:** Tsinghua-Berkeley Shenzhen Institute (TBSI), Tsinghua University, Shenzhen 518000, China; clr18@mails.tsinghua.edu.cn (L.C.); maosm19@mails.tsinghua.edu.cn (S.M.); li-z19@mails.tsinghua.edu.cn (Z.L.); sherry_fto@outlook.com (Y.H.)

In the original publication [1], there was a mistake in *Equation (8)* as published. Given the angle definition in the publication, “cosθ” in *Equation (8)* should be “sinθ”. The corrected *Equation (8)* is shown below.

(8) $$m\lambda =n_{eff}\sqrt{x^{2}+y^{2}}-xn_{0}\sin\theta$$

The authors apologize for any inconvenience caused and state that the scientific conclusions are unaffected. This correction was approved by the Academic Editor. The original publication has also been updated.
